# Body Composition Is Related to Maximal Effort Treadmill Test Time in Firefighters

**DOI:** 10.3390/healthcare11111607

**Published:** 2023-05-31

**Authors:** Benjamin J. Mendelson, Rudi A. Marciniak, Carly A. Wahl, Kyle T. Ebersole

**Affiliations:** 1 Human Performance & Sport Physiology Laboratory, Department of Rehabilitation Sciences & Technology, University of Wisconsin-Milwaukee, Milwaukee, WI 53211, USA; 2 Department of Kinesiology, Sport, & Recreation, Eastern Illinois University, Charleston, IL 61920, USA

**Keywords:** firefighter, body composition, aerobic capacity, occupational health, occupational performance

## Abstract

Firefighting tasks may require near maximal levels of cardiorespiratory fitness. Previous research has indicated that body fat percentage (BF%) and aerobic capacity (VO_2peak_) are related to the performance of firefighting tasks. Since a standard submaximal treadmill test for firefighters is terminated at 85% of maximal heart rate (MHR), key performance information relating to maximal cardiorespiratory effort may not be measured in a submaximal test. The purpose of this study was to examine the relationships between body composition and time spent running at intensities greater that 85% MHR. Height, weight, body mass index (BMI; kg/m^2^), BF%, MHR (bpm), VO_2peak_ (mL/kg/min), predicted VO_2peak_ (P-VO_2peak_; mL/kg/min), submaximal treadmill test time (WFI_sub_ Test Time; min), and maximal treadmill test time (WFI_max_ Test Time; min) were collected in fifteen active-duty firefighters. The results indicated that significant relationships (*p* < 0.05) existed between BF% and VO_2peak_, BF% and WFI_max_ Test Time, BF% and T_diff_, and VO_2peak_ and WFI_max_ Test Time. P-VO_2peak_ was not significantly different than VO_2peak_, and the WFI_max_ Test Time was significantly longer than the WFI_sub_ Test Time. These results indicate that a submaximal treadmill test may reasonably predict VO_2peak_, but key information about physiological work at intensities greater than 85% MHR may be missed when using submaximal effort tests.

## 1. Introduction

Firefighting is widely recognized as dangerous work that requires a high amount of exertion [1]. As such, it has been reported that high levels of physical fitness are beneficial for overall firefighter performance [2]. Firefighter performance has been previously quantified by the time to complete job-specific tasks, such as forcible entry, victim rescues, hose pulls, and ladder climbs, among other duties required of a firefighter [2,3,4,5,6,7,8]. It has also been reported that firefighting job tasks may require oxygen consumption rates between 16.8–44.0 mL/kg/min [8]. The combination of physical fitness qualities (e.g., muscular strength, muscular endurance, aerobic capacity) to complete job tasks while using the specific equipment of firefighting (i.e., tools, personal protective equipment) has been previously attributed to the terms “work capacity” [9,10], “work ability” [11,12], or “physiological capacity” [13]. Work capacity presents insight into methods of improving performance and decreasing risk of injury, such as improving physical fitness. Even with the National Fire Protection Association (NFPA) recommendation of a minimum aerobic capacity (VO_2peak_) of 42.0 mL/kg/min [14] to safely complete job tasks, it was reported that 31 firefighters died due to sudden cardiac death in 2021, accounting for 44% of total annual firefighter fatalities that year [15]. This raises considerations for firefighter work ability and work capacity as a possible measure to better understand the cardiovascular demands of firefighting.

The joint effort by the International Association of Firefighters and International Association of Fire Chiefs Wellness Fitness Initiative (WFI) outlined a submaximal treadmill test to estimate maximal aerobic capacity [16]. This submaximal assessment of VO_2peak_ is terminated at 85% predicted maximal heart rate (MHR). This submaximal assessment approach has merit for the ability to predict VO_2peak_ without a maximal effort test [16,17]; however, the submaximal test just estimates and does not measure the ability to work at maximal or near maximal intensities. Gaesser and Poole [18] identified three exercise domains (i.e., moderate, heavy, severe) that correspond to increasing levels of an internal load response from moderate to severe, including increases in HR and oxygen consumption (VO_2_). The moderate and heavy domains represent work intensities in which a steady state can be attained, while the severe domain represents work at intensities where VO_2_ and blood lactate are not able to stabilize. This indicates that exercise in the severe intensity domain does not reach a steady state, and prolonged exercise in the severe domain leads to increases in oxygen consumption and blood lactate until fatigue [18,19]. Mielke et al. [20] and Bergstrom et al. [21] examined the critical heart rate, a measure of the heart rate intensity at which a person crosses from heavy to severe exercise, and reported that critical heart rate may range between 83–94% MHR. Interestingly, this range aligns with the 85% MHR benchmark at which the WFI submaximal protocol is terminated, which suggests that the time difference between the termination of the WFI submaximal protocol and maximal exertion may represent the severe intensity domain. Thus, it is possible that the use of submaximal test protocols does not allow for an opportunity to fully understand the workability of a firefighter in the severe intensity domain (i.e., >85% MHR).

Furthermore, the body composition of a firefighter may influence the ability to perform at high aerobic intensity levels. Previous research has indicated firefighter performance and absenteeism due to an injury is associated with higher levels of obesity as measured by body mass index (BMI) [22]. However, a known limitation of BMI, a standard value of obesity, is that firefighters may be misclassified as obese when their body composition (i.e., body fat percentage) does not align with obesity [22,23]. Regardless, prior literature is generally in agreement that high levels of percent body fat may negatively impact the expression of muscle strength or aerobic capacity by contributing to an earlier onset of fatigue [24,25], thereby decreasing the time a person can perform work. Thus, it is possible that body composition may influence the ability of a firefighter to perform work in the severe intensity domain. Therefore, the purpose of this study was to examine the relationships between body fat percentage, VO_2peak_, and the time a firefighter was able to spend running at a heart rate > 85% MHR to maximal effort.

## 2. Materials and Methods

### 2.1. Participants and Study Design

Participants in this study included 15 active-duty firefighters from an urban fire department (13 male and 2 female; see Table 1 for participant characteristics). This study was conducted in accordance with the Declaration of Helsinki, and all study protocols were approved by the Institutional Review Board at the University of Wisconsin-Milwaukee (Protocol Number: 19.A.197, Approved on 28 February 2019), with all participants providing written informed consent.

### 2.2. Anthropometric Data

Anthropometric data were collected at the beginning of the laboratory testing session, including height measured to the nearest 0.01 cm, weight in kilograms (kg), and BMI (kg/m^2^). Body fat percentage (BF%) was estimated from skinfold measures using the Three Skinfold Site Jackson and Pollock Method [26]. Skinfold sites included subscapular, tricep, and pectoral for male participants, and tricep, abdominal and suprailiac for female participants. Each site was measured twice and measured to the nearest millimeter (mm) of thickness using a handheld skinfold caliper (Beta Technology, Inc., Santa Cruz, CA, USA). If the two measurements at a given site differed by ≥2mm, the site was measured a third time and the average of all three measurements was taken. All skinfolds were measured by the same researcher.

### 2.3. Aerobic Capacity Testing

Participants completed a treadmill test using the protocol defined by the International Association of Firefighters and International Association of Fire Chiefs WFI protocol on a motorized treadmill (Woodway USA Inc., Waukesha, WI, USA). The test started at 3.0 mph for three minutes at a 0% grade incline. After the initial three minutes, the velocity was increased to 4.5 mph. Stages then progressed in 1-min intervals, alternating between increases in grade (2%) or velocity (0.5 mph), until maximal effort was exerted (WFI_max_). The WFI_max_ test was terminated when at least two of the following three criteria were achieved: (a) meeting or exceeding predicted MHR (MHR = 208 − 0.7 × age) for more than 15 s, (b) participant rating of perceived exertion was ≥17 on Borg’s 6–20 scale, and/or (c) volitional termination by the participant due to fatigue [27]. Expired air was measured via gas analysis (Fitmate MED, COSMED, Rome, Italy) to accurately measure maximal oxygen consumption during the test (VO_2peak_; mL/kg/min). The total time of the WFI_max_ treadmill test was also recorded (WFI_max_ Test Time; min). Heart rate was monitored continuously throughout the maximal treadmill test via a Zephyr Bioharness 3 wireless physiological status monitor (Medtronic, Annapolis, MD, USA) at a sampling rate of 250 Hz to capture MHR achieved during the treadmill test.

The submaximal version of the WFI protocol (WFI_sub_) was terminated when the participant achieved 85% of age-predicted maximal heart rate using the following equation:

$$85\%\;\mathrm{Predicted}\;\mathrm{MHR}=208-\left(0.7\times \;\mathrm{age}\;\left(\mathrm{years}\right)\right)\times 0.85$$

The time at which the participant achieved criteria for the submaximal test during the maximal test was recorded and used post hoc in the WFI submaximal treadmill VO_2peak_ prediction equation to calculate for comparison to WFI_max_:

$$P-{\mathrm{VO}}_{2\mathrm{peak}}=56.981+\left(1.242\times {\;\mathrm{WFI}}_{\mathrm{sub}}\;\mathrm{Test}\;\mathrm{Time}\right)-\left(0.805\times \mathrm{BMI}\right)$$

The time difference in minutes between the WFI_sub_ and WFI_max_ was calculated (T*_diff_*) for each participant and used as a relative comparison to severe intensity domain of exercise, as the results from Bergstrom et al. [21] indicated that the critical heart rate may range between 83–94% of MHR.

### 2.4. Statistical Analysis

Paired-samples *t*-tests were used to determine significant mean differences between predicted MHR and MHR achieved on the treadmill test, WFI_sub_ Test Time and WFI_max_ Test Time, and P-VO_2peak_ and achieved VO_2peak_. Additionally, Pearson-product correlations were used to investigate significant relationships between BF%, VO_2peak_, achieved MHR, WFI_max_ Test Time, WFI_sub_ Test Time, and T_diff_. Correlation coefficients were interpreted using the following guidelines/parameters [28]: very weak: <0.20, weak: <0.20–0.39, moderate: 0.40–0.59, strong: 0.60–0.79, or very strong: >0.80. Statistical significance was determined with an alpha-level of *p* ≤ 0.05. Data were analyzed using Statistical Package for the Social Sciences (v. 28.0.1.1; IBM SPSS Inc., Chicago, IL, USA).

## 3. Results

Table 1 provides descriptive statistics for all variables. The results of the paired samples *t*-test indicated no significant difference between predicted MHR and MHR achieved during the maximal treadmill test (*t*_14_ = −1.386, *p* = 0.187), as well as no significant difference between P-VO_2peak_ and VO_2peak_ (*t*_14_ = −0.796, *p* = 0.439). However, the WFI_max_ Test Time was significantly longer than the WFI_sub_ Test Time (*t*_14_ = −22.039, *p* < 0.001). All participants volitionally terminated the test after meeting at least two of the three criteria for termination on the maximal treadmill test.

Results of the Pearson-product correlations (Table 2) indicated moderate to strong significant negative relationships between BF% and VO_2peak_ (r = −0.762, *p* < 0.001), WFI_max_ Test Time (r = −0.704, *p* < 0.001), and T_diff_ (r = −0.636, *p* < 0.05). In addition, there were strong to very strong positive significant relationships between the VO_2peak_ and WFI_sub_ Test Time (r = 0.711, *p* < 0.001) and the WFI_max_ Test Time (r = 0.863, *p* < 0.001).

## 4. Discussion

### 4.1. Submaximal WFI Treadmill Test

Submaximal aerobic capacity testing has long been used in many populations due to the ability to estimate VO_2peak_ safely and in a relatively short amount of time [29,30]. In the fire service, the WFI submaximal treadmill test was developed to provide fire departments with a method to easily and reliably predict VO_2peak_. However, the literature has been mixed regarding the ability of the WFI treadmill test to predict VO_2peak_ with some researchers reporting stronger predictability [17] and other researchers reporting significant differences [27,29,31] between the WFI VO_2peak_ estimate and the actual VO_2peak_ measured from a maximal test. Accordingly, it has been suggested that methodological differences may account for the equivocal findings in the literature. For example, multiple studies have assessed the WFI submaximal protocol’s ability to predict VO_2peak_ when compared to maximal protocols, including the WFI protocol carried to maximal exertion [17,29,32], constant treadmill speed with increasing grade [31], Bruce Maximal Protocol [27], and other maximal treadmill tests [30]. Mier et al. [31] suggested that the inaccuracy present in the prediction of the maximal heart rate using the equation 220—(age) and the use of the ACSM metabolic equation for running to predict VO_2peak_, which is primarily used for steady state running, may skew the predictability of the WFI equation to overpredict VO_2peak_. The results of this study, however, indicated that the VO_2peak_ estimated from the submaximal WFI treadmill test was not significantly different from the VO_2peak_ directly measured from the maximal treadmill test.

The current study used protocols similar to Dolezal et al. [29], Klaren et al. [32], and Drew-Nord et al. [17], in which a single test was performed and the time point at which a participant met the submaximal 85%MHR termination point of the WFI submaximal test was noted, but the participant continued to perform the test to maximal ability. It is interesting to note, however, that the VO_2peak_ estimates in the current study are similar to those reported in other studies using a firefighter population [27,28,29,31,33,34]. Thus, the results of the current study indicate that the WFI submaximal treadmill protocol may be an accurate prediction of VO_2peak_, yet the impact of terminating the test at 85% MHR may leave out critical performance-related information.

### 4.2. Maximal Treadmill Test

**Figure 1:**
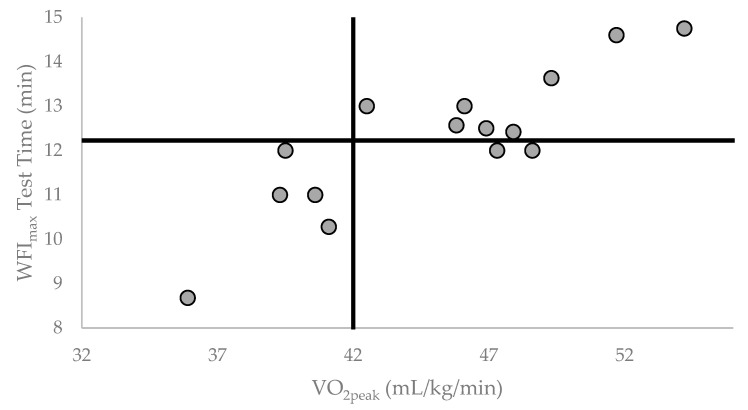
Individual participant WFI_max_ Test Time (min) by VO_2peak_ (mL/kg/min). Vertical reference line placed at the NFPA recommended VO_2peak_ = 42.0 mL/kg/min. Horizontal reference line placed at group mean for WFI_max_ Test Time = 12.23 min.

**Figure 2:**
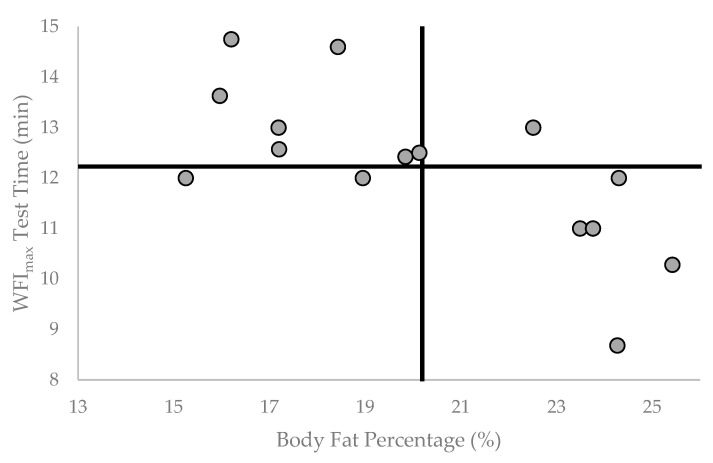
Individual participant WFI_max_ Test Time (min) by Body Fat % (BF%). Vertical reference line is set at group mean BF% = 20.19. Horizontal Reference line is set at group mean WFI_max_ Test Time = 12.23 min.

Fire suppression calls generally demand a firefighter to function at a high level of intensity for a sustained time period, requiring near maximal levels of heart rate and oxygen consumption [34,35,36,37]. Sothmann et al. [36] reported that the mean responses to actual fire suppression emergencies was 88 ± 6% MHR and 63 ± 14% of VO_2peak_ across an average duration of 15 ± 7 min for an emergency call. In this study, the maximal treadmill test elicited a MHR response of 101 ± 4% of age predicted MHR and lasted 12.23 ± 1.58 min which, as expected, was longer (4.69 ± 0.82 min) than the submaximal test termination time point. The results of this study indicated that a lower total time on the treadmill during a maximal test was significantly associated with lower VO_2peak_ (Figure 1) and higher BF% (Figure 2). Interestingly, the significant negative relationship between BF% and T_diff_ (*p* < 0.05) would suggest that BF% is related specifically to the time on the treadmill in the severe HR intensity domain (i.e., >85% MHR). Thus, the ability of a firefighter to work in the severe HR intensity domain, which is often observed during a fire suppression task, may be related to VO_2peak_ and BF%.

Previous research has indicated that VO_2peak_ is a significant predictor of various measures of firefighter performance, including that VO_2peak_ is a predictor of how quickly a firefighter will deplete the air tank in the self-contained breathing apparatus (SCBA) [38]. Additionally, lower VO_2peak_ has been previously linked to a slower performance on job-specific ability tasks [39,40,41]. Williford et al. [4] indicated that the time to complete a 1.5-mile run had a moderate, but significant, relationship (*r* = 0.38, *p* < 0.001) to the total time to complete a job-specific ability test. Interestingly, Rhea et al. [3] found that the distance covered in a Cooper’s 12-min run was not significantly related (*r* = −0.32, *p* > 0.05) to the time to complete a job-specific ability test, but that anaerobic endurance (i.e., time to complete a 400-m run) was significantly related (*r =* 0.79, *p* < 0.05) to time to complete the job-specific ability test, suggesting that the ability to perform high intensity anaerobic activities may play a role in firefighter performance.

**Figure 3:**
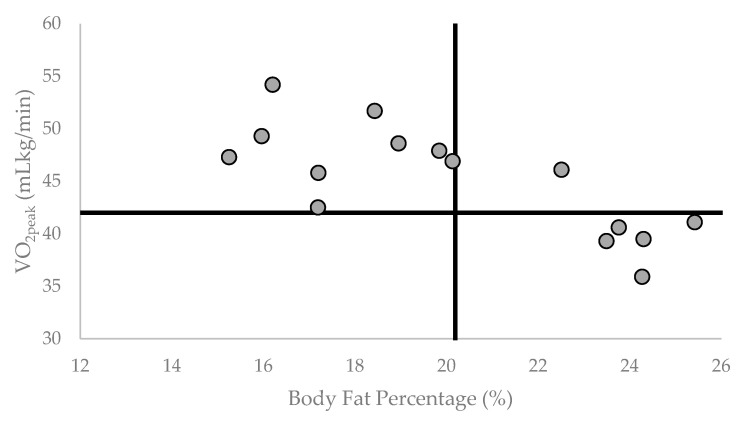
Individual participant Body Fat % (BF%) by VO_2peak_ (mL/kg/min). Vertical reference line is set at group mean BF% = 20.19. Horizontal Reference line is set at NFPA recommended VO_2peak_ = 42.0 mL/kg/min.

To date, several studies have investigated the relationship between body composition and aerobic capacity, but there is limited information relating those characteristics to the total test time on a maximal treadmill test in firefighter populations. Storer et al. [42] conducted a comprehensive fitness and cardiovascular risk screening in a sample of active-duty firefighters. In that study, it was reported that BF% and VO_2peak_ measured from either an incremental treadmill walking, running, or cycle protocol had a significant negative association (*p* = 0.003), but they did not report relationships between BF% or VO_2peak_ and test time. Kiss et al. [43] also reported that BF% had a significant relationship with VO_2peak_ (*r* = −0.61, *p* < 0.001) in a sample of Belgian firefighters, but did not report on relationships between BF% or VO_2peak_ and treadmill test time. McKinney et al. [44] indicated that BF% accounted for 58% of the variation in VO_2peak_ measured by treadmill in a sample of active-duty firefighters, but a relationship between BF% and test time was not reported. Lastly, Norris et al. [33] found a significant relationship between VO_2peak_ and treadmill time to exhaustion (*r* = 0.93, *p* < 0.01) in active-duty firefighters, but they did not report relationships between BF% and VO_2peak_ or treadmill test time. Thus, the results of the present study agree with previous studies that have found significant negative relationships between BF% and VO_2peak_ (Figure 3) and build upon the previous research by indicating that a time-based performance outcome (i.e., time of exercise > 85% MHR) may be reduced in the presence of higher BF% in firefighters.

While these results provide insight into the ability of firefighters to work at intensities >85% MHR, there are limitations to be considered for future research. Despite the sample being reflective of the average percentage of male and female career firefighters in the United States [45], the sample size was small. Therefore, future researchers should seek to replicate these procedures with a larger sample size. Additionally, previous research has suggested that a potential mechanism for BF% to negatively impact time-based performance in firefighters is by acting as non-functional mass that contributes to increased neuromuscular fatigue [25,46,47]. Previous studies have linked higher neuromuscular output (i.e., greater muscular strength, muscular endurance, and anaerobic power) to faster time to complete simulated job-specific ability tests in firefighters [3,4,6,25,33] and higher BF% to slower time to complete tasks [4,5,6] individually. Recently, it has been reported that higher BF% was related to lower neuromuscular output and greater fatigability during an isotonic leg extension task [48], increased time to complete a stair climb [48], and decreased muscular endurance [49,50] in firefighters. However, there were no neuromuscular output variables involved in this study. Future research should investigate the relationships between neuromuscular output, body composition, aerobic capacity, and work time in a maximal treadmill test to further understand this mechanism.

## 5. Conclusions

These results suggest that BF% and VO_2peak_ may be important performance factors to severe intensity domain (i.e., >85% MHR) work in firefighters. A diminished ability to work in the severe intensity domain may present as a slower time to complete firefighting tasks [6,25,40] or a faster SCBA air depletion time [38], which may impact the optimal ability to perform job tasks. Thus, submaximal protocols may reliably predict VO_2peak_, yet they may omit key performance information about maximal intensity work.

## Figures and Tables

**Table 1:** Descriptive statistics for anthropometric and treadmill data.

	Mean ± SD	Range
Age (yrs)	35.27 ± 8.28	22.00–49.00
Height (m)	1.79 ± 0.07	1.70–1.91
Weight (kg)	89.14 ± 16.09	64.18–122.56
BMI (kg/m^2^)	27.64 ± 4.76	22.00–39.90
Body Fat (%)	20.19 ± 3.49	15.25–25.42
Max HR (bpm)	186.0 ± 9.65	168.00–199.00
P-VO_2peak_ (mL/kg/min)	44.09 ± 4.61	33.43–49.35
VO_2peak_ (mL/kg/min)	45.11 ± 5.12	35.90–54.20
WFI_sub_ Test Time (min)	7.54 ± 1.38	4.83–10.75
WFI_max_ Test Time (min)	12.23 ± 1.58	8.68–14.75
T_diff_ (min)	4.69 ± 0.82	3.38–6.27

**Table 2:** Relationships among anthropometric, aerobic capacity, and treadmill data.

	VO_2peak_	MHR	WFI_sub_ Test Time	WFI_max_ Test Time	T_diff_
BF%	−0.762 **	0.053	−0.426	−0.704 **	−0.636 *
VO_2peak_		−0.093	0.711 **	0.863 **	0.462
MHR			−0.173	−0.085	0.127
WFI_sub_ Test Time				0.853 **	−0.040
WFI_max_ Test Time					0.487

* = significant at 0.05 level. ** = significant at 0.01 level.

## Data Availability

The data presented in this study are available on request from the corresponding author.
